# Predictive model for managing the clinical risk of emergency department patients: protocol for a systematic review

**DOI:** 10.1136/bmjhci-2025-101584

**Published:** 2025-12-11

**Authors:** Maria João Baptista Rente, Ana Lúcia da Silva João, David José Murteira Mendes, Liliana Andreia Neves da Mota

**Affiliations:** 1 Comprehensive Health Research Centre, Universidade de Évora, Palácio dos Colegiais 2, 7004-516, Évora, Portugal; 2 Serviço de Urgência Médico-Cirúrgico, Departamento de Urgência e Emergência, Unidade Local de Saúde do Litoral Alentejano, EPE, Monte do Gilbardinho, 7540-230, Santiago do Cacém, Setúbal, Portugal; 3 Enfermagem, Escola Superior de Saúde de Santarém, Instituto Politécnico de Santarém, Quinta do Mergulhão, Srª da Guia, 2005-075, Santarém, Portugal; 4 Conselho Técnico-Científico, Escola Superior de Saúde Norte da Cruz Vermelha Portuguesa, Rua da Cruz Vermelha Cidacos—Apartado 1002, 3720-126, Oliveira de Azeméis, Aveiro, Portugal; 5 LT3 - Ciência de Dados, de Decisão & Tecnologias de Informação, Tech4edusim—Technologies for Education and Simulation in Healthcare, CINTESIS: Centro de Investigação em Tecnologias e Serviços de Saúde, Escola Superior de Enfermagem do Porto, Rua Dr. António Bernardino de Almeida, 830, 844, 856, 4200-072, Porto, Portugal

**Keywords:** Artificial intelligence, Data Science, Decision Support Systems, Management, Emergency Service, Hospital, Deep Learning

## Abstract

**Introduction:**

Emergency departments are facing increasing strain due to overcrowding and resource shortages, leading to the suspension of some services. Stratifying the clinical risk—defined as the severity and likelihood of harm—is crucial for anticipating care needs and supporting decision-making. Implementing predictive models for clinical risk management offers a technological solution to this challenge. This systematic review will evaluate the performance and usefulness of a predictive model for managing the clinical risk of people who visit the emergency department.

**Methods and analysis:**

Eight electronic databases will be searched (CINAHL Plus, Health Technology Assessment Database, MedicLatina, MEDLINE, PubMed, Scopus, Cochrane Plus Collection, Web of Science). Risk of bias will be assessed using the Checklist for Critical Appraisal and Data Extraction for Systematic Reviews of Prediction Modelling Studies and Prediction Model Risk of Bias Assessment Tool.

**Ethics and dissemination:**

Ethical approval is not required. Results will be disseminated through peer-reviewed publications.

**PROSPERO registration number:**

CRD42024556926.

**WHAT IS ALREADY KNOWN ON THIS TOPIC**

- The increase in expected waiting times contributes to a deterioration in the person’s state of health, with a consequent increase in clinical risk, with a significant impact on the provision of care.
- Emergency health professionals who often race against time to save lives, emphasising the importance of their work for society.
- Emergency healthcare professionals are the perfect union of technological expertise and compassion, ensuring that life is a priority every second of the way.

**WHAT THIS STUDY ADDS**

- This systematic review will provide evidence on the effects of a predictive clinical risk management model on people who visit the emergency department.
- This study will examine the strength of the causal association between a predictive clinical risk management model and emergency department outcomes among people who visit the department.
- This study will provide evidence about the feasibility of a predictive clinical risk management model in emergency departments, in terms of anticipating care needs and informing decision-making.

**HOW THIS STUDY MIGHT AFFECT RESEARCH,PRACTICE OR POLICY**

- The study will positively affect research due to the lack of evidence on standardised predictive models for clinical risk management of emergency department patients, hence the importance of studying this topic.
- The study will include various types of interventions for managing the clinical risk of people who visit the emergency department to assess the performance and usefulness of a predictive model for managing the clinical risk of people who use the emergency department, which will positively affect practice.
- A predictive model for clinical risk management will enable emergency healthcare professionals to systematise their actions and care management, based on transdisciplinary decision-making, with a view to the health and well-being of the person, fulfilling the objectives of the policy.

## Introduction

 The high demand for emergency departments has placed significant strain on national health services, contributing to overcrowding, long waiting times and reduced efficiency.^1 2^

International strategies have emphasised the need to strengthen emergency response and patient safety. The WHO European Programme of Work 2020–2025 and the Thirteenth General Programme of Work prioritise protecting people better against health emergencies, ensuring healthy lives and well-being.^3^

Similarly, the Global Patient Safety Action Plan 2021–2030 aims to eliminate avoidable harm by improving the quality and safety of healthcare and achieving the Sustainable Development Goals.^4^

These efforts align with the United Nations 2030 Agenda for Sustainable Development, particularly the third goal, Quality Health, which calls for accessible, high-quality healthcare and strengthened capacities for risk management.^5^

Nationally, the Basic Health Law in Base 1 states that personal safety is one of the fundamental dimensions/components of the right to health protection.^6^ The National Patient Safety Plan 2021–2026 aims to consolidate and promote safety in the provision of healthcare.^7^ Emergency departments are crucial access points for urgent care, making early identification and management of clinical risk essential to reduce morbidity and mortality.^8 9^

Nursing research adopts a holistic and transdisciplinary approach to understanding health and illness. The nursing process integrates ethical and scientific knowledge to inform clinical practice.^10 11^

Predictive models, based on artificial intelligence algorithms, support clinicians by analysing large datasets to assess clinical risk, enabling faster and more tailored decision-making.^12^

Nurses frequently assume responsibility for risk assessment, particularly during triage, where they are often the first point of contact.^13^,^15^ While decision-support tools are available,^16^ nurses’ clinical judgement remains essential.^13 14^

Recent systematic reviews have explored machine learning applications in emergency departments, including triage systems,^17 18^ deterioration prediction^18 19^ and hospital admission forecasting.^18 20^ Broader applications of artificial intelligence in emergency department risk stratification have also been examined.^21^ However, few studies focus on models specifically designed for overall clinical risk management, which involves complex interactions between patient characteristics, clinical indicators and service conditions.^20 22^

Predictive models, whether statistical or artificial intelligence driven, aim to predict deterioration and support decision-making throughout the care pathway.^23^ Despite their potential, issues around performance, validation and implementation persist.^12^ A focused review is needed to evaluate these models considering clinical, safety and informatics goals.

As new technologies emerge, predictive models must evolve accordingly.^22^ Given the complexity of clinical risk, such tools are increasingly vital for planning and delivering safe, high-quality care.^24^

For this review, clinical risk refers to the potential for harm to a patient, based on clinical indicators, severity and likelihood of deterioration.^13 14^ Predictive models are defined as algorithm-based tools—often derived from machine learning or statistical techniques—that estimate this risk to support decision-making in emergency settings.^25 26^

The approach proposed for this protocol aims to carry out a systematic review to evaluate the performance and usefulness of a predictive model for managing the clinical risk of people who visit the emergency department.

The question the review will address is: What are the predictive models for managing the clinical risk of people used in emergency departments?

## Methods

This protocol was registered in PROSPERO (CRD42024556926) and was reported according to the guidelines of the Preferred Reporting Items for Systematic Review and Meta-Analysis (PRISMA)Protocols (online supplemental Appendix A).^27 28^ Additionally, this systematic review will be conducted following the PRISMA guideline.^29 30^ The systematic review will be conducted in accordance with the recommendations of the Cochrane Handbook for Systematic Reviews of Interventions.^31^

### Eligibility criteria

The population, intervention, comparison and outcome approach will be used to select the studies (table 1).

**Table 1:** PICO-SD framework

PICO-SD framework	Elements	Description
Population	People aged 18 and over, not pregnant, who use the emergency department	–
Intervention	Clinical risk management in emergency departments	Clinical risk management refers to the process of minimising liability exposure in healthcare settings by focusing on safety, security and quality of patient care.^13 14^
Comparison	Early warning score	Early warning scores are simple tools to help detect clinical deterioration to improve patient safety in hospitals.^19^
Outcome	Predictive model	Predictive models are machine learning models which are trained to analyse historical data to find patterns and trends, allowing them to predict future outcomes.^25 26^ Due to the exploratory scope and inclusive approach of this review, predictive models will be eligible regardless of whether they have undergone internal or external validation. However, the validation status of each model will be recorded and discussed.
Study design	We will include original peer-reviewed primary research studies, including both randomised controlled trials and observational studies (eg, cohort, case–control, cross-sectional designs). Study protocols, conference abstracts, dissertations/theses and trial registry records will also be considered. We will exclude publication types that do not provide original primary data—such as systematic reviews, narrative reviews, editorials, commentaries, letters to the editor, consensus statements, books/book chapters and erratum/corrections.	Original peer-reviewed primary research studies are suitable for assessment using the GRADE approach.^33 34^ Study protocols, conference abstracts, dissertations/theses and trial registry records will enhance comprehensiveness and reduce publication bias. These sources will be carefully appraised for completeness and quality. Excluded publication types are not suitable for GRADE-based evaluation.^33 34^ However, reference lists from relevant reviews may be screened for eligible studies.
Year and language	Articles in Portuguese, Spanish and English will be included, with no limit on publication time.	Only studies published in English, Portuguese or Spanish will be included. This may limit the comprehensiveness of the review, but reflects the language capabilities of the review team.

GRADE, Grading of Recommendations Assessment, Development and Evaluation.

### Information sources

Electronic databases: CINAHL Plus, Health Technology Assessment Database, MedicLatina, MEDLINE, PubMed, Scopus, Cochrane Plus Collection, Web of Science.

This review will include only peer-reviewed articles. Grey literature and preprints will be excluded to ensure data quality and methodological rigour.

The selection of electronic databases was based on the Cochrane Handbook for Systematic Reviews of Interventions: Chapter 4: Searching for and selecting studies.^31^ MedicLatina was selected because it is a collection of scientific and medical research journals from renowned Latin American and Spanish publishers, peer-reviewed in Spanish.

### Search strategy

The research will include the combination of six key concepts according to Medical Subject Headings (table 2).

**Table 2:** Medical Subject Headings terms

Terms	Entry terms
Risk Assessment	Health Risk Assessment, Risk Analysis
Risk Management	–
Risk Adjustment	Case-Mix Adjustment
Risk Factors	–
Early Warning Score	–
Emergency Service, Hospital	Emergency Departments

The strategy will be adapted according to each database (online supplemental Appendix B).

### Study records

Data management: The mechanism that will be used to manage records and data throughout the review is a web-based tool for organising, managing and accelerating collaborative systematic literature reviews named Rayyan.

**Figure 1:**
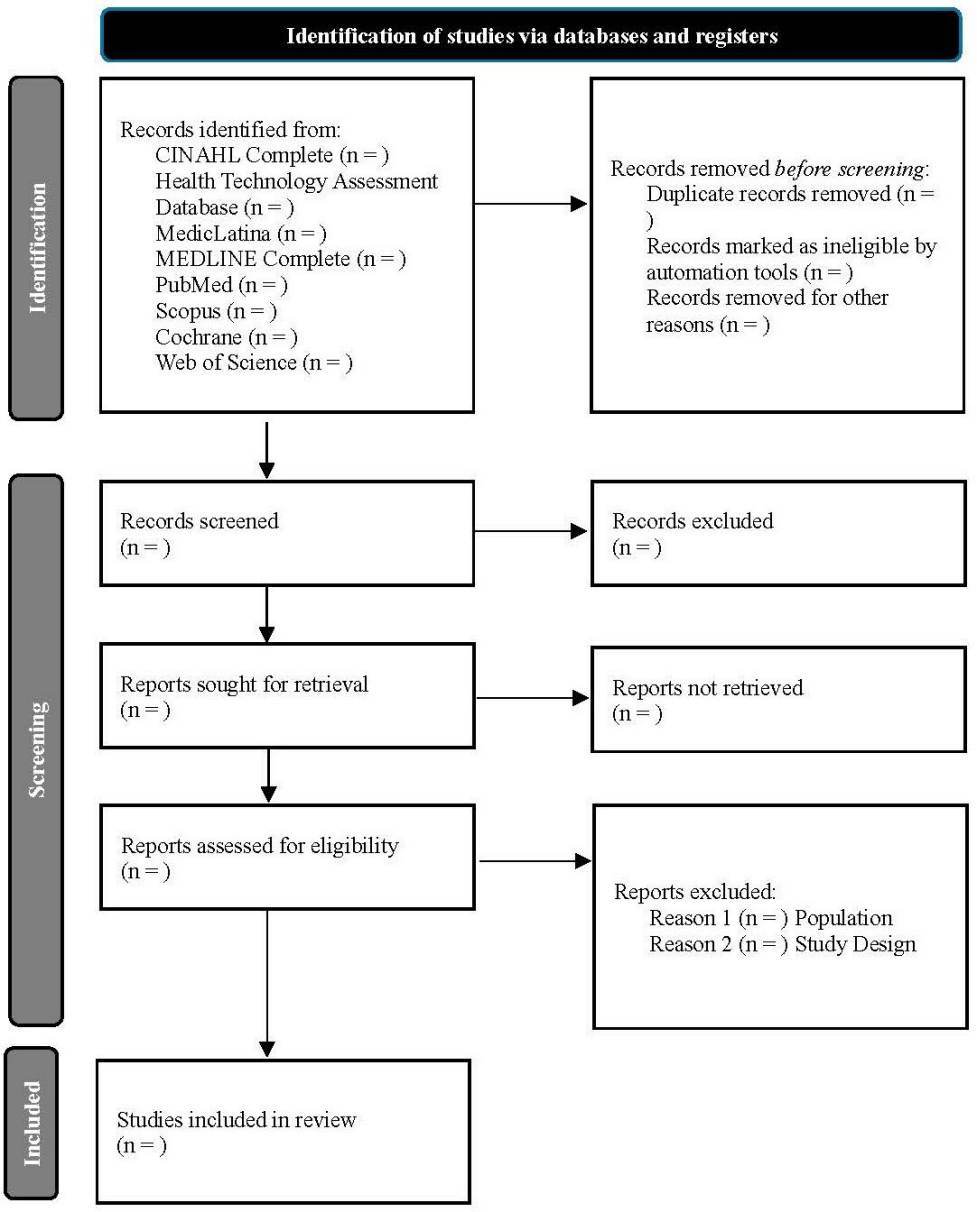
PRISMA flow diagram of selection process. PRISMA, Preferred Reporting Items for Systematic Review and Meta-Analysis.

Selection process: Two authors will independently review all identified studies from the literature searches. In the first phase, unsuitable studies will be excluded by reviewing titles and abstracts. All studies identified as relevant by either of the reviewers will be checked in full text. In the second phase, the full text of the remaining studies will be read and classified as include or exclude. The reasons for exclusion will be obtained. In case of doubts or disagreement, a third reviewer will be consulted. A PRISMA flow diagram (figure 1)^29^ will be used to illustrate the selection process.

Data collection process: The data extraction and analysis will follow the guidelines of the Cochrane Handbook of Systematic Reviews of Interventions (table 3).^31^

**Table 3:** Data collection process

Study details	Authors, publication year, language, country where the study was carried out, study aim/research question, design, recruitment source, inclusion and exclusion criteria, type of allocation, stratification, sample size.
Characteristics of participants	Age, gender, ethnicity, multimorbidities.
Intervention details	Intervention content, intervention setting, delivery of intervention, number of participants assessed at follow-up points.
Comparison/control characteristics	Type of control programme/interventions.
Outcomes	Primary and secondary outcomes (between-group differences, total scores (mean) and SD in each group will be extracted from the study results, methods of outcome measurement including blinding procedures, time of outcome measurements.

### Data items

The following data items will be extracted from each included study using a standardised data extraction form, guided by the Cochrane Handbook of Systematic Reviews of Interventions (table 4).^31^

**Table 4:** Data items

Study characteristics	Author(s), year of publication, language, country, research objective, study design, recruitment setting/source, inclusion and exclusion criteria, type of allocation or stratification, and sample size.
Population characteristics	Age (≥18 years), gender, ethnicity (if reported) and presence of multimorbidities. Studies involving pregnant individuals will be excluded.
Intervention characteristics	Description and content of the clinical risk management intervention, intervention setting (eg, hospital emergency department), delivery mode and number of participants followed over time.
Comparison/control characteristics	Type and details of comparator, specifically early warning scores.
Outcome measures	Type and definition of the outcome, performance metrics (eg, sensitivity, specificity, area under the curve), methods of measurement, blinding procedures (if applicable) and timing of outcome assessment.

All extracted data will be entered into a table and cross-checked by a second reviewer. Disagreements will be resolved by discussion or adjudication by a third reviewer.

### Outcomes and prioritisation

The primary outcome of interest is the performance and utility of predictive models used for clinical risk management in emergency departments. This includes metrics such as accuracy, sensitivity, specificity, positive/negative predictive value, area under the curve and calibration measures.

Secondary outcomes may include comparative performance with standard early warning scores.

The review will prioritise outcomes that reflect both predictive performance and clinical applicability of the models in real-world emergency department settings. The inclusion of clinical utility of the model in supporting real-time decision-making, impact on patient care processes (eg, timeliness, triage accuracy) and implementation feasibility or integration into practice, will enable a comprehensive synthesis of outcome data across study designs and contexts.

### Risk of bias in individual studies

Two review authors will independently assess the risk of bias in included studies using the CHARMS (Checklist for Critical Appraisal and Data Extraction for Systematic Reviews of Prediction Modelling Studies) and PROBAST (Prediction Model Risk of Bias Assessment Tool).^32^ Any disagreements will be resolved by consensus, or if needed, by a third review author. The risk of bias assessment for each study will be presented in a table along with other characteristics of the included studies.

### Data synthesis

A table will be built to summarise the data summary and the answers to the research questions, which will group the characteristics of the included studies: authors; year; sample; objectives; assessment tools; interventions; results; conclusions.

This form of representation will make it possible to group and synthesise the data collected from each study and will facilitate the analysis and discussion of the results.

The summary of results will be reported in narrative text to facilitate the comparison of the results of each study included in the review and a description of the results will be made identifying the studies.

Heterogeneity across model types will be addressed through a narrative synthesis. Particular attention will be given to inconsistencies in results—such as divergent effect directions or substantial variations in effect magnitude—across studies. A meta-analysis will not be conducted due to the anticipated methodological and clinical heterogeneity.

### Meta-bias(es)

The assessment of meta-biases will include checking for selective reporting within studies by comparing reported outcomes with those listed in study protocols (when available). In addition, although a formal meta-analysis will not be conducted, the potential for publication bias will be considered by noting the presence or absence of non-significant findings and by documenting whether studies were registered in advance (eg, in clinical trial registries). Discrepancies between protocols and final publications will be critically appraised and discussed.

### Confidence in cumulative evidence

The overall certainty of evidence for each outcome will be assessed using the Grading of Recommendations Assessment, Development and Evaluation (GRADE) approach framework (table 5), following guidance from the GRADE Handbook^33 34^ and recent literature.^35^

**Table 5:** GRADE assessment for each outcome will be rated as high, moderate, low or very low certainty

GRADE criteria	Description
Risk of bias	Will be assessed using appropriate tools (eg, CHARMS and PROBAST)^32^ and will lead to downgrading where methodological limitations such as inadequate blinding, missing data, model overfitting or selective reporting are prevalent across studies.^33^,^35^
Inconsistency	Will result in downgrading when substantial heterogeneity exists in the direction or magnitude of effect estimates, which cannot be explained by subgroup analyses or differences in study design. We will consider heterogeneity both statistically and clinically.^33^,^35^
Indirectness	Will be considered when the population, intervention, comparator or outcomes differ meaningfully from the research question—for example, studies conducted in different care settings or populations than those specified in our protocol.^33^,^35^
Imprecision	Will be judged based on the width of CIs and total information size. We will downgrade if CIs cross thresholds of clinical importance or if the sample size is insufficient to draw robust conclusions, particularly regarding model performance metrics like discrimination or calibration.^33^,^35^
Publication bias	Will be evaluated by examining funnel plot asymmetry, trial registries, grey literature and other sources of unpublished data. Where publication bias is suspected, certainty will be downgraded.^33^,^35^

CHARMS, Checklist for Critical Appraisal and Data Extraction for Systematic Reviews of Prediction Modelling Studies; GRADE, Grading of Recommendations Assessment, Development and Evaluation; PROBAST, Prediction Model Risk of Bias Assessment Tool.

This approach will ensure a transparent and structured assessment of the strength of the evidence base underpinning our review findings.

## Supplementary material

10.1136/bmjhci-2025-101584 online supplemental file 1
