# Supplementary material for: Predictive model for managing the clinical risk of emergency department patients: protocol for a systematic review
Source: BMJ Health Care Inform. 2025 Dec 11;32(1):e101584. doi: 10.1136/bmjhci-2025-101584 (PMC12699717; doi:10.1136/bmjhci-2025-101584)
Supplement: online supplemental file 1 [file bmjhci-32-1-s001.pdf]

Supplemental Material

Appendix A – PRISMA-P (Preferred Reporting Items for Systematic review and Meta-Analysis Protocols) 2015 checklist: recommended items to address in a systematic review protocol*

Section and topic	Item No	Checklist item	Page and Line
**ADMINISTRATIVE INFORMATION**			
Title:			Page 1, Line 8 to 14
Identification	1a	Identify the report as a protocol of a systematic review	Page 1, Line 12
Update	1b	If the protocol is for an update of a previous systematic review, identify as such	Page 1, Line 14
Registration	2	If registered, provide the name of the registry (such as PROSPERO) and registration number	Page 1, Line 19
Authors:			Page 1, Line 23 to 59<br>Page 2, Line 3 to 5
Contact	3a	Provide name, institutional affiliation, e-mail address of all protocol authors; provide physical mailing address of corresponding author	Page 1, Line 26 to 48
Contributions	3b	Describe contributions of protocol authors and identify the guarantor of the review	Page 1, Line 50 to 59<br>Page 2, Line 3 to 5
Amendments	4	If the protocol represents an amendment of a previously completed or published protocol, identify as such and list changes; otherwise, state plan for documenting important protocol amendments	Page 2, Line 9
Support:			Page 2, Line 14 to 21
Sources	5a	Indicate sources of financial or other support for the review	Page 2, Line 16
Sponsor	5b	Provide name for the review funder and/or sponsor	Page 2, Line 18
Role of sponsor or funder	5c	Describe roles of funder(s), sponsor(s), and/or institution(s), if any, in developing the protocol	Page 2, Line 21
**INTRODUCTION**			
Rationale	6	Describe the rationale for the review in the context of what is already known	Page 4, Line 3 to 59<br>Page 5, Line 3 to 31
Objectives	7	Provide an explicit statement of the question(s) the review will address with reference to participants, interventions, comparators, and outcomes (PICO)	Page 5, Line 33 and 40

**METHODS**			
Eligibility criteria	8	Specify the study characteristics (such as PICO, study design, setting, time frame) and report characteristics (such as years considered, language, publication status) to be used as criteria for eligibility for the review	Page 6, Line 3 to 46<br>Page 6, Table 1
Information sources	9	Describe all intended information sources (such as electronic databases, contact with study authors, trial registers or other grey literature sources) with planned dates of coverage	Page 6, Line 50 to 57<br>Page 7, Line 3 to 10
Search strategy	10	Present draft of search strategy to be used for at least one electronic database, including planned limits, such that it could be repeated	Page 7, Line 15 to 28<br>Page 7, Table 2
Study records:			Page 7 to 8
Data management	11a	Describe the mechanism(s) that will be used to manage records and data throughout the review	Page 7, Line 34 to 39
Selection process	11b	State the process that will be used for selecting studies (such as two independent reviewers) through each phase of the review (that is, screening, eligibility and inclusion in meta-analysis)	Page 7, Line 41 to 53
Data collection process	11c	Describe planned method of extracting data from reports (such as piloting forms, done independently, in duplicate), any processes for obtaining and confirming data from investigators	Page 7, Line 55 to 57<br>Page 8, Table 3
Data items	12	List and define all variables for which data will be sought (such as PICO items, funding sources), any pre-planned data assumptions and simplifications	Page 8, Line 17 to 38<br>Page 8, Table 4
Outcomes and prioritization	13	List and define all outcomes for which data will be sought, including prioritization of main and additional outcomes, with rationale	Page 8, Line 42 to 59<br>Page 9, Line 3 to 7
Risk of bias in individual studies	14	Describe anticipated methods for assessing risk of bias of individual studies, including whether this will be done at the outcome or study level, or both; state how this information will be used in data synthesis	Page 9, Line 11 to 22
Data synthesis	15a	Describe criteria under which study data will be quantitatively synthesised	Page 9, Line 26 to 49
	15b	If data are appropriate for quantitative synthesis, describe planned summary measures, methods of handling data and methods of combining data from studies, including any planned exploration of consistency (such as I^2, Kendall's τ)	Page 9, Line 26 to 49
	15c	Describe any proposed additional analyses (such as sensitivity or subgroup analyses, meta-regression)	Page 9, Line 26 to 49
	15d	If quantitative synthesis is not appropriate, describe the type of summary planned	Page 9, Line 26 to 49
Meta-bias(es)	16	Specify any planned assessment of meta-bias(es) (such as publication bias across studies, selective reporting within studies)	Page 9, Line 54 to 59<br>Page 10, Line 3 to 9
Confidence in cumulative evidence	17	Describe how the strength of the body of evidence will be assessed (such as GRADE)	Page 10, Line 12 to 38<br>Page 10, Table 5

* It is strongly recommended that this checklist be read in conjunction with the PRISMA-P Explanation and Elaboration (cite when available) for important clarification on the items. Amendments to a review protocol should be tracked and dated. The copyright for PRISMA-P (including checklist) is held by the PRISMA-P Group and is distributed under a Creative Commons Attribution Licence 4.0.

From: Shamseer L, Moher D, Clarke M, Ghersi D, Liberati A, Petticrew M, Shekelle P, Stewart L, PRISMA-P Group. Preferred reporting items for systematic review and meta-analysis protocols (PRISMA-P) 2015: elaboration and explanation. BMJ. 2015 Jan 2;349(jan02 1):g7647.

Appendix B – Complete Syntaxes

CINAHL® Plus

(risk assessment OR Risk Assessments OR Assessment, Risk OR Health Risk Assessment OR Assessment, Health Risk OR Health Risk Assessments OR Risk Assessment, Health OR Risk Analysis OR Analysis, Risk OR Risk Analyses OR risk management OR Management, Risk OR Management, Risks OR Risks Management OR risk adjustment OR Adjustment, Risk OR Adjustments, Risk OR Risk Adjustments OR Case-Mix Adjustment OR Adjustment, Case-Mix OR Adjustments, Case-Mix OR Case Mix Adjustment OR Case-Mix Adjustments OR risk factors OR Factor, Risk OR Risk Factor) AND (early warning score OR Early Warning Scores OR Score, Early Warning OR Scores, Early Warning OR Warning Scores, Early) AND (emergency service, hospital OR Emergency Services, Hospital OR Hospital Emergency Services OR Services, Hospital Emergency OR Hospital Emergency Service OR Service, Hospital Emergency OR Emergency Hospital Service OR Emergency Hospital Services OR Hospital Service, Emergency OR Hospital Services, Emergency OR Service, Emergency Hospital OR Services, Emergency Hospital OR Hospital Service Emergency OR Emergencies, Hospital Service OR Emergency, Hospital Service OR Hospital Service Emergencies OR Service Emergencies, Hospital OR Service Emergency, Hospital OR Emergency Departments OR Department, Emergency OR Departments, Emergency OR Emergency Department)

Health Technology Assessment Database

((risk assessment) OR (Risk Assessments) OR (Assessment, Risk) OR (Health Risk Assessment) OR (Assessment, Health Risk) OR (Health Risk Assessments) OR (Risk Assessment, Health) OR (Risk Analysis) OR (Analysis, Risk) OR (Risk Analyses) OR (risk management) OR (Management, Risk) OR (Management, Risks) OR (Risks Management) OR (risk adjustment) OR (Adjustment, Risk) OR (Adjustments, Risk) OR (Risk Adjustments) OR (Case-Mix Adjustment) OR (Adjustment, Case-Mix) OR (Adjustments, Case-Mix) OR (Case Mix Adjustment) OR (Case-Mix Adjustments) OR (risk factors) OR (Factor, Risk) OR (Risk Factor)) AND ((early warning score) OR (Early Warning Scores) OR (Score, Early Warning) OR (Scores, Early Warning) OR (Warning Scores, Early)) AND ((emergency service, hospital) OR (Emergency Services, Hospital) OR (Hospital Emergency Services) OR (Services, Hospital Emergency) OR (Hospital

Emergency Service) OR (Service, Hospital Emergency) OR (Emergency Hospital Service) OR (Emergency Hospital Services) OR (Hospital Service, Emergency) OR (Hospital Services, Emergency) OR (Service, Emergency Hospital) OR (Services, Emergency Hospital) OR (Hospital Service Emergency) OR (Emergencies, Hospital Service) OR (Emergency, Hospital Service) OR (Hospital Service Emergencies) OR (Service Emergencies, Hospital) OR (Service Emergency, Hospital) OR (Emergency Departments) OR (Department, Emergency) OR (Departments, Emergency) OR (Emergency Department))

MedicLatina

(risk assessment OR Risk Assessments OR Assessment, Risk OR Health Risk Assessment OR Assessment, Health Risk OR Health Risk Assessments OR Risk Assessment, Health OR Risk Analysis OR Analysis, Risk OR Risk Analyses OR risk management OR Management, Risk OR Management, Risks OR Risks Management OR risk adjustment OR Adjustment, Risk OR Adjustments, Risk OR Risk Adjustments OR Case-Mix Adjustment OR Adjustment, Case-Mix OR Adjustments, Case-Mix OR Case Mix Adjustment OR Case-Mix Adjustments OR risk factors OR Factor, Risk OR Risk Factor) AND (early warning score OR Early Warning Scores OR Score, Early Warning OR Scores, Early Warning OR Warning Scores, Early) AND (emergency service, hospital OR Emergency Services, Hospital OR Hospital Emergency Services OR Services, Hospital Emergency OR Hospital Emergency Service OR Service, Hospital Emergency OR Emergency Hospital Service OR Emergency Hospital Services OR Hospital Service, Emergency OR Hospital Services, Emergency OR Service, Emergency Hospital OR Services, Emergency Hospital OR Hospital Service Emergency OR Emergencies, Hospital Service OR Emergency, Hospital Service OR Hospital Service Emergencies OR Service Emergencies, Hospital OR Service Emergency, Hospital OR Emergency Departments OR Department, Emergency OR Departments, Emergency OR Emergency Department)

MEDLINE®

(risk assessment OR Risk Assessments OR Assessment, Risk OR Health Risk Assessment OR Assessment, Health Risk OR Health Risk Assessments OR Risk Assessment, Health OR Risk Analysis OR Analysis, Risk OR Risk Analyses OR risk management OR Management, Risk OR Management, Risks OR Risks Management OR risk adjustment

OR Adjustment, Risk OR Adjustments, Risk OR Risk Adjustments OR Case-Mix Adjustment OR Adjustment, Case-Mix OR Adjustments, Case-Mix OR Case Mix Adjustment OR Case-Mix Adjustments OR risk factors OR Factor, Risk OR Risk Factor) AND (early warning score OR Early Warning Scores OR Score, Early Warning OR Scores, Early Warning OR Warning Scores, Early) AND (emergency service, hospital OR Emergency Services, Hospital OR Hospital Emergency Services OR Services, Hospital Emergency OR Hospital Emergency Service OR Service, Hospital Emergency OR Emergency Hospital Service OR Emergency Hospital Services OR Hospital Service, Emergency OR Hospital Services, Emergency OR Service, Emergency Hospital OR Services, Emergency Hospital OR Hospital Service Emergency OR Emergencies, Hospital Service OR Emergency, Hospital Service OR Hospital Service Emergencies OR Service Emergencies, Hospital OR Service Emergency, Hospital OR Emergency Departments OR Department, Emergency OR Departments, Emergency OR Emergency Department)

PubMed

(((((((((((((((((((((((((((risk assessment) OR (Risk Assessments)) OR (Assessment, Risk)) OR (Health Risk Assessment)) OR (Assessment, Health Risk)) OR (Health Risk Assessments)) OR (Risk Assessment, Health)) OR (Risk Analysis)) OR (Analysis, Risk)) OR (Risk Analyses)) OR (risk management)) OR (Management, Risk)) OR (Management, Risks)) OR (Risks Management)) OR (risk adjustment)) OR (Adjustment, Risk)) OR (Adjustments, Risk)) OR (Risk Adjustments)) OR (Case-Mix Adjustment)) OR (Adjustment, Case-Mix)) OR (Adjustments, Case-Mix)) OR (Case Mix Adjustment)) OR (Case-Mix Adjustments)) OR (risk factors)) OR (Factor, Risk)) OR (Risk Factor)) AND (((((early warning score) OR (Early Warning Scores)) OR (Score, Early Warning)) OR (Scores, Early Warning)) OR (Warning Scores, Early))) AND ((((((((((((((((((((((emergency service, hospital) OR (Emergency Services, Hospital)) OR (Hospital Emergency Services)) OR (Services, Hospital Emergency)) OR (Hospital Emergency Service)) OR (Service, Hospital Emergency)) OR (Emergency Hospital Service)) OR (Emergency Hospital Services)) OR (Hospital Service, Emergency)) OR (Hospital Services, Emergency)) OR (Service, Emergency Hospital)) OR (Services, Emergency Hospital)) OR (Hospital Service Emergency)) OR (Emergencies, Hospital Service)) OR (Emergency, Hospital Service)) OR (Hospital Service Emergencies)) OR (Service Emergencies, Hospital)) OR (Service Emergency, Hospital)) OR (Emergency

Departments)) OR (Department, Emergency)) OR (Departments, Emergency)) OR (Emergency Department))

Scopus

TITLE-ABS-KEY (risk AND assessment) OR (risk AND assessments)) OR (assessment, AND risk)) OR (health AND risk AND assessment)) OR (assessment, AND health AND risk)) OR (health AND risk AND assessments)) OR (risk AND assessment, AND health)) OR (risk AND analysis)) OR (analysis, AND risk)) OR (risk AND analyses)) OR (risk AND management)) OR (management, AND risk)) OR (management, AND risks)) OR (risks AND management)) OR (risk AND adjustment)) OR (adjustment, AND risk)) OR (adjustments, AND risk)) OR (risk AND adjustments)) OR (case-mix AND adjustment)) OR (adjustment, AND case-mix)) OR (adjustments, AND case-mix)) OR (case AND mix AND adjustment)) OR (case-mix AND adjustments)) OR (risk AND factors)) OR (factor, AND risk)) OR (risk AND factor)) AND (((((early AND warning AND score) OR (early AND warning AND scores)) OR (score, AND early AND warning)) OR (scores, AND early AND warning)) OR (warning AND scores, AND early))) AND (emergency AND service, AND hospital) OR (emergency AND services, AND hospital)) OR (hospital AND emergency AND services)) OR (services, AND hospital AND emergency)) OR (hospital AND emergency AND service)) OR (service, AND hospital AND emergency)) OR (emergency AND hospital AND service)) OR (emergency AND hospital AND services)) OR (hospital AND service, AND emergency)) OR (hospital AND services, AND emergency)) OR (service, AND emergency AND hospital)) OR (services, AND emergency AND hospital)) OR (hospital AND service AND emergency)) OR (emergencies, AND hospital AND service)) OR (emergency, AND hospital AND service)) OR (hospital AND service AND emergencies)) OR (service AND emergencies, AND hospital)) OR (service AND emergency, AND hospital)) OR (emergency AND departments)) OR (department, AND emergency)) OR (departments, AND emergency)) OR (emergency AND department)))

Cochrane Plus Collection

(risk assessment OR Risk Assessments OR Assessment, Risk OR Health Risk Assessment OR Assessment, Health Risk OR Health Risk Assessments OR Risk Assessment, Health

OR Risk Analysis OR Analysis, Risk OR Risk Analyses OR risk management OR Management, Risk OR Management, Risks OR Risks Management OR risk adjustment OR Adjustment, Risk OR Adjustments, Risk OR Risk Adjustments OR Case-Mix Adjustment OR Adjustment, Case-Mix OR Adjustments, Case-Mix OR Case Mix Adjustment OR Case-Mix Adjustments OR risk factors OR Factor, Risk OR Risk Factor) AND (early warning score OR Early Warning Scores OR Score, Early Warning OR Scores, Early Warning OR Warning Scores, Early) AND (emergency service, hospital OR Emergency Services, Hospital OR Hospital Emergency Services OR Services, Hospital Emergency OR Hospital Emergency Service OR Service, Hospital Emergency OR Emergency Hospital Service OR Emergency Hospital Services OR Hospital Service, Emergency OR Hospital Services, Emergency OR Service, Emergency Hospital OR Services, Emergency Hospital OR Hospital Service Emergency OR Emergencies, Hospital Service OR Emergency, Hospital Service OR Hospital Service Emergencies OR Service Emergencies, Hospital OR Service Emergency, Hospital OR Emergency Departments OR Department, Emergency OR Departments, Emergency OR Emergency Department)

Web of Science

((((((((((((((((((((((((((ALL=(Risk Assessment)) OR ALL=(Risk Assessments)) OR ALL=(Assessment, Risk)) OR ALL=(Health Risk Assessment)) OR ALL=(Assessment, Health Risk)) OR ALL=(Health Risk Assessments)) OR ALL=(Risk Assessment, Health)) OR ALL=(Risk Analysis)) OR ALL=(Analysis, Risk)) OR ALL=(Risk Analyses)) OR ALL=(risk management)) OR ALL=(Management, Risk)) OR ALL=(Management, Risks)) OR ALL=(Risks Management)) OR ALL=(risk adjustment)) OR ALL=(Adjustment, Risk)) OR ALL=(Adjustments, Risk)) OR ALL=(Risk Adjustments)) OR ALL=(Case-Mix Adjustment)) OR ALL=(Adjustment, Case-Mix)) OR ALL=(Adjustments, Case-Mix)) OR ALL=(Case Mix Adjustment)) OR ALL=(Case-Mix Adjustments)) OR ALL=(risk factors)) OR ALL=(Factor, Risk)) OR ALL=(Risk Factor)) AND (((((ALL=(early warning score)) OR ALL=(Early Warning Scores)) OR ALL=(Score, Early Warning)) OR ALL=(Scores, Early Warning)) OR ALL=(Warning Scores, Early)) AND ((((((((((((((((((((((ALL=(emergency service, hospital)) OR ALL=(Emergency Services, Hospital)) OR ALL=(Hospital Emergency Services)) OR ALL=(Services, Hospital Emergency)) OR ALL=(Hospital Emergency Service)) OR ALL=(Service, Hospital Emergency)) OR ALL=(Emergency Hospital

Service)) OR ALL=(Emergency Hospital Services)) OR ALL=(Hospital Service, Emergency)) OR ALL=(Hospital Services, Emergency)) OR ALL=(Service, Emergency Hospital)) OR ALL=(Services, Emergency Hospital)) OR ALL=(Hospital Service Emergency)) OR ALL=(Emergencies, Hospital Service)) OR ALL=(Emergency, Hospital Service)) OR ALL=(Hospital Service Emergencies)) OR ALL=(Service Emergencies, Hospital)) OR ALL=(Service Emergency, Hospital)) OR ALL=(Emergency Departments)) OR ALL=(Department, Emergency)) OR ALL=(Departments, Emergency)) OR ALL=(Emergency Department))
